# Metabolites of an Oil Field Sulfide-Oxidizing, Nitrate-Reducing *Sulfurimonas* sp. Cause Severe Corrosion

**DOI:** 10.1128/AEM.01891-18

**Published:** 2019-01-23

**Authors:** Sven Lahme, Dennis Enning, Cameron M. Callbeck, Demelza Menendez Vega, Thomas P. Curtis, Ian M. Head, Casey R. J. Hubert

**Affiliations:** a School of Natural and Environmental Sciences, Newcastle University, Newcastle upon Tyne, United Kingdom; b ExxonMobil Upstream Research Company, Spring, Texas, USA; c Max Planck Institute for Marine Microbiology, Bremen, Germany; d School of Engineering, Newcastle University, Newcastle upon Tyne, United Kingdom; e Department of Biological Sciences, University of Calgary, Calgary, Alberta, Canada; Wageningen University

**Keywords:** microbiologically influenced corrosion, nitrate reduction, oil field microbiology, souring control, sulfide oxidation

## Abstract

Oil reservoir souring and associated material integrity challenges are of great concern to the petroleum industry. The bioengineering strategy of nitrate injection has proven successful for controlling souring in some cases, but recent reports indicate increased corrosion in nitrate-treated produced water reinjection facilities. Sulfide-oxidizing, nitrate-reducing bacteria (soNRB) have been suggested to be the cause of such corrosion. Using the model soNRB Sulfurimonas sp. strain CVO obtained from an oil field, we conducted a detailed analysis of soNRB-induced corrosion at initial nitrate-to-sulfide (N/S) ratios relevant to oil field operations. The activity of strain CVO caused severe corrosion rates of up to 0.27 millimeters per year (mm y^−1^) and up to 60-μm-deep pitting within only 9 days. The highest corrosion during the growth of strain CVO was associated with the production of zero-valent sulfur during sulfide oxidation and the accumulation of nitrite, when initial N/S ratios were high. Abiotic corrosion tests with individual metabolites confirmed biogenic zero-valent sulfur and nitrite as the main causes of corrosion under the experimental conditions. Mackinawite (FeS) deposited on carbon steel surfaces accelerated abiotic reduction of both sulfur and nitrite, exacerbating corrosion. Based on these results, a conceptual model for nitrate-mediated corrosion by soNRB is proposed.

**IMPORTANCE** Ambiguous reports of corrosion problems associated with the injection of nitrate for souring control necessitate a deeper understanding of this frequently applied bioengineering strategy. Sulfide-oxidizing, nitrate-reducing bacteria have been proposed as key culprits, despite the underlying microbial corrosion mechanisms remaining insufficiently understood. This study provides a comprehensive characterization of how individual metabolic intermediates of the microbial nitrogen and sulfur cycles can impact the integrity of carbon steel infrastructure. The results help explain the dramatic increases seen at times in corrosion rates observed during nitrate injection in field and laboratory trials and point to strategies for reducing adverse integrity-related side effects of nitrate-based souring mitigation.

## Abstract (precis)

Ambiguous reports of corrosion problems associated with the injection of nitrate for souring control necessitate a deeper understanding of this frequently applied bioengineering strategy. Sulfide-oxidizing, nitrate-reducing bacteria have been proposed as key culprits, despite the underlying microbial corrosion mechanisms remaining insufficiently understood. This study provides a comprehensive characterization of how individual metabolic intermediates of the microbial nitrogen and sulfur cycles can impact the integrity of carbon steel infrastructure. The results help explain the dramatic increases seen at times in corrosion rates observed during nitrate injection in field and laboratory trials and point to strategies for reducing adverse integrity-related side effects of nitrate-based souring mitigation.

## INTRODUCTION

Petroleum reservoirs and their associated facilities for processing and transporting oil, gas, and water represent vast engineered ecosystems that harbor a large diversity of microorganisms. Biofilm formation on internal metal surfaces in pipelines and other equipment can lead to microbiologically influenced corrosion (MIC). Sulfate-reducing, fermentative, acetogenic, methanogenic, metal-reducing, and metal-oxidizing microorganisms have all been implicated in the degradation of steel in the oil and gas industry (1–9).

Another microbiological phenomenon that affects oil field operations is reservoir souring, i.e., the production of hydrogen sulfide (H_2_S) by sulfidogenic microorganisms in water-flooded reservoirs. In reservoir souring, the causative microorganisms and the infrastructure they affect can be many kilometers apart from each other. Biogenic H_2_S from the reservoir is carried in produced fluids and gases to production facilities where it can exacerbate corrosion. More importantly, H_2_S can lead to catastrophic failures of carbon steel well tubing and other high-pressure equipment through sulfide stress cracking (10).

Reservoir souring is difficult to control through chemical injection due to the expanse and remoteness of the biologically active zones within reservoirs (11). One approach that is applied on a commercial scale is the continuous injection of nitrate into a reservoir, typically at concentrations between 0.8 and 4 mM (50 to 250 ppm). This bioengineering strategy has proven successful in multiple laboratory experiments (12–15). Under the much-less-controlled conditions in oil field-wide nitrate applications, mixed accounts have been given regarding the efficacy of nitrate injection (16, 17), highlighting the need for a better understanding of the variables governing its success.

Nitrate is delivered to the reservoir via seawater or recycled produced water that is injected in large volumes for secondary oil recovery (17). In recent years, operators have observed increased corrosion in produced water reinjection (PWRI) facilities to which nitrate was added for souring control purposes (18–20). Laboratory studies have similarly detected increased corrosion in experiments with mixed microbial communities and nitrate addition (21–23). The formation of zero-valent sulfur (S^0^), an intermediate of sulfide-oxidizing, nitrate-reducing bacteria (soNRB), has been hypothesized to be responsible for such corrosion (19, 21, 23), given that S^0^ is known as a strong oxidant of iron (24–27). Other mechanistic explanations of MIC under nitrate-reducing conditions have also been offered, including the formation of nitrite, consumption of cathodic hydrogen, direct electron uptake from Fe^0^, and sulfur disproportionation (18–23).

Epsilonproteobacterial soNRB are frequently detected in oil reservoirs and production systems (28–30) and have been identified in corrosive biofilms during PWRI tests with nitrate (18, 20). These microorganisms can accumulate S^0^ depending on the ratio of nitrate to sulfide (31).

(1) $$\begin{array}{cc} {\text{HS}}^{-}+1.6\;{\text{NO}}_{3}^{-}+0.6\;{\text{H}}^{+}\to {\text{SO}}_{4}^{2-}+0.8\;{\text{N}}_{2}+0.8\;{\text{H}}_{2}\text{O}, & \Delta {\text{G}}^{0\prime }=-748.3\;{\text{kJ mol}}^{-1} \end{array}$$

(2) $$\begin{array}{ll} {\text{HS}}^{-}+0.4\;{\text{NO}}_{3}^{-}+1.4\;{\text{H}}^{+}\to {\text{S}}^{0}+0.2\;{\text{N}}_{2}+1.2\;{\text{H}}_{2}\text{O}, & \Delta {\text{G}}^{0\prime }=-197.2\;{\text{kJ mol}}^{-1} \end{array}$$

Complete oxidation of sulfide to sulfate is expected at nitrate-to-sulfide (N/S) ratios of ≥1.6 (equation 1), whereas incomplete oxidation and accumulation of potentially corrosive S^0^ are expected at N/S ratios of <1.6, especially with limiting quantities of nitrate (N/S ratio, ≤0.4; equation 2). On the other hand, excess nitrate (N/S ratio, ≥1.6) likely results in the accumulation of nitrite (equation 3).

(3) $$\begin{array}{ll} {\text{HS}}^{-}+4\;{\text{NO}}_{3}^{-}\to {\text{SO}}_{4}^{2-}+4\;{\text{NO}}_{2}^{-}+{\text{H}}^{+}, & \Delta {\text{G}}^{0\prime }=-510.4\;{\text{kJ mol}}^{-1} \end{array}$$

Therefore, we hypothesized that the N/S ratio could have an effect on corrosion due to the accumulation of different soNRB metabolites.

In this study, the soNRB isolate Sulfurimonas sp. strain CVO (32, 33), obtained from an oil field, was used to investigate MIC under the controlled conditions of pure-culture experiments at varied N/S ratios; Sulfurimonas spp., including strain CVO, have been shown to form a major part of the microbial community during oil-field-wide nitrate injection (28, 33, 34). We first generated technically relevant corrosion rates in the laboratory and studied nitrate-mediated MIC through the comprehensive characterization of sulfur and nitrogen transformations in corrosion experiments. We then elucidated the cause of MIC in these systems through experimental dissection of the individual chemical reactions of the biologically formed intermediates with metallic iron (Fe^0^). Based on the results, a conceptual model for soNRB-mediated corrosion is proposed.

## RESULTS

### Corrosion associated with different N/S ratios.

**FIG 1:**
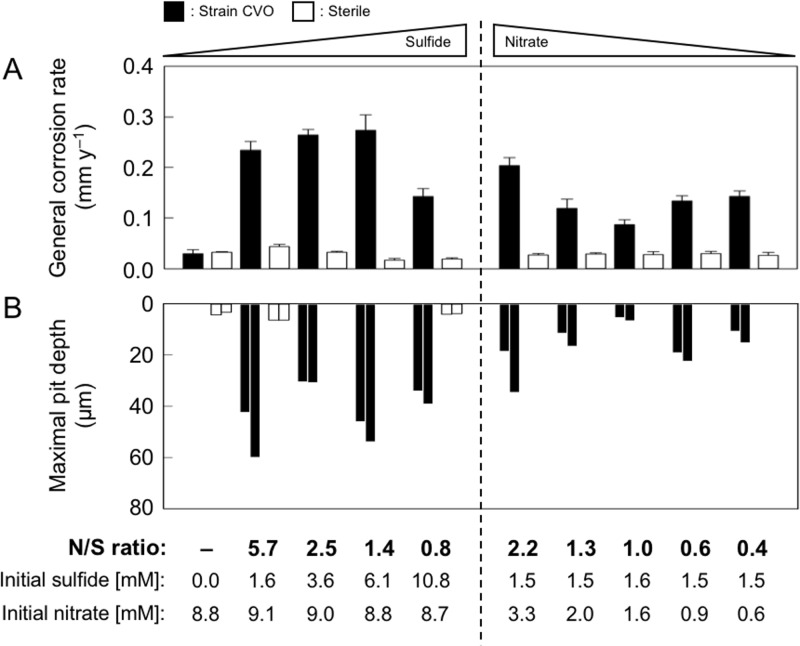
Corrosion of carbon steel coupons after 9 days exposure to cultures of *Sulfurimonas* sp. strain CVO or sterile medium with various initial levels of sulfide and nitrate (i.e., different nitrate-to-sulfide [N/S] ratios). (A) Average general corrosion rates determined by weight loss of 1-cm by 1-cm carbon steel coupons. Error bars represent the standard deviation from the results from nine coupons from triplicate incubations. (B) Maximum pit depth after 9 days measured on duplicate X52 carbon steel coupons.

To test the effect of the accumulation of different S and N metabolites on corrosion, carbon steel coupons were exposed to growing cultures of strain CVO at different initial N/S ratios (Fig. 1; see also Table S1 in the supplemental material).

Iron supplied to CVO cultures with initial N/S ratios between 0.4 and 5.7 corroded at rates between 0.09 and 0.27 millimeters per year (mm y^−1^), whereas sterile sulfidic or sulfide-free incubations resulted in corrosion rates of ≤0.04 mm y^−1^ (Fig. 1A). The highest rates of 0.20 to 0.27 mm y^−1^ were observed at N/S ratios of ≥1.4. Lower ratios (≤1.3) resulted in corrosion rates approximately 50% lower, with the lowest rate of 0.09 mm y^−1^ observed at an N/S ratio of 1.0.

**FIG 2:**
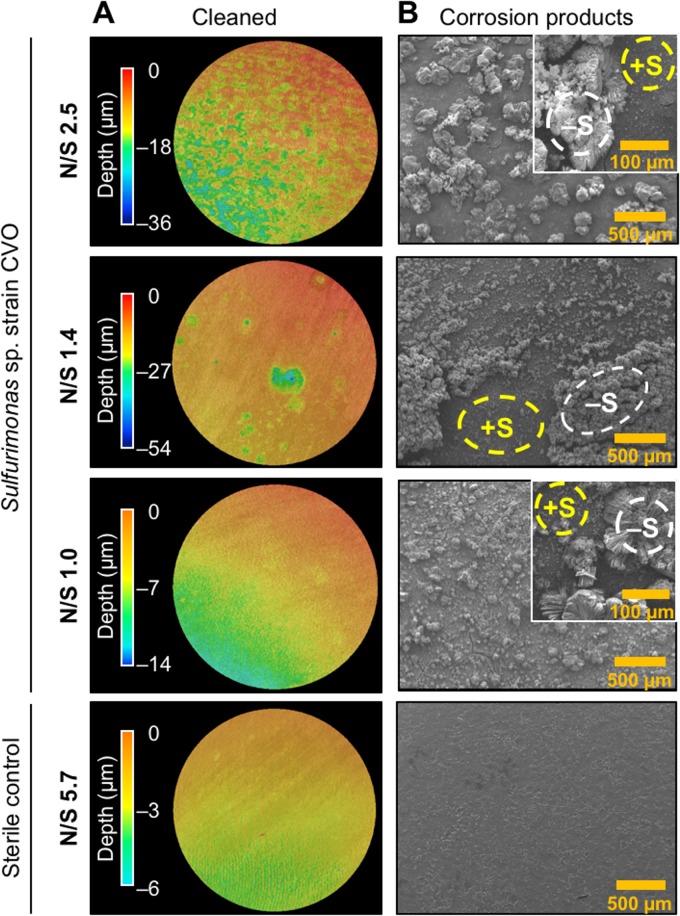
(A) Images obtained by white-light interferometry showing pitting corrosion of X52 carbon steel coupons (corrosion products removed). The color scale indicates depth of individual pits. (B) Energy-dispersive X-ray (EDX) microanalysis of corrosion crust deposited on the surface of carbon steel coupons used for weight loss corrosion analysis. High sulfur content (+S, yellow) was detected on the surface and was absent or markedly reduced in mineral structures (–S, white).

In addition, corrosion caused by strain CVO was also characterized by white-light interferometry to assess pitting, which reached up to 60 μm depth in 9-day experiments, compared to only 6 μm in sterile controls (Fig. 1B, 2A, and S1). Pitting and general corrosion (weight loss) rates followed a similar trend with respect to N/S ratios, i.e., deeper pits (19 to 60 μm) were more frequently associated with excess nitrate (N/S ratio, ≥1.4), whereas lower nitrate doses (N/S ratio, ≤1.3) gave rise to maximum pit depths of only 5 to 22 μm, with the least pitting observed at an N/S ratio of 1.0.

### Corrosion products formed by soNRB activity.

Minerals formed on corroding metal surfaces can provide mechanistic insights into underlying MIC reactions. Scanning electron micrographs showed the formation of mineral structures on corroded coupons in all CVO cultures, regardless of the N/S ratio (Fig. 2B and S2) but not in sterile controls. Semiquantitative energy-dispersive X-ray (EDX) spot analysis of corrosion crusts from CVO cultures identified sulfur (7 to 26 [wt%] S; Table S2) on flat surfaces between largely sulfur-free mineral structures (<5 [wt%] S). Combined oxygen and nitrogen (47 to 55 [wt%] O+N) and phosphorus (8 to 12 [wt%] P) signals were also elevated in all detected mineral structures. X-ray diffraction analysis (XRD) identified surface-associated vivianite [Fe_3_(PO_4_)_2_·8H_2_O] in CVO cultures, whereas mackinawite (FeS) was identified as the main surface-associated product in sulfidic sterile controls (Fig. S3). Together, EDX and XRD data suggest that the mineral deposits consisted mainly of vivianite, whereas the sulfur-rich surfaces consisted mainly of the iron sulfide mackinawite.

### Sulfur and nitrogen chemistry in soNRB cultures.

**FIG 3:**
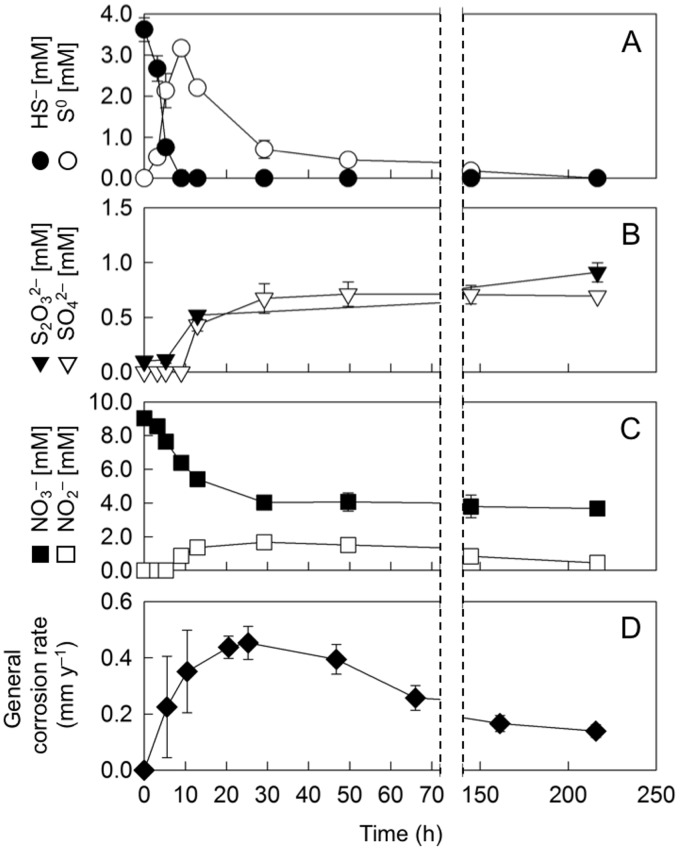
(A to C) Concentration changes of sulfur and nitrogen species in cultures of strain CVO at a nitrate-to-sulfide (N/S) ratio of 2.5. (D) Corrosion rate time series in cultures of *Sulfurimonas* sp. strain CVO at an N/S ratio of 2.5. See Fig. S5 for weight loss profile and sterile controls.

Sulfide-oxidizing nitrate-reducing bacteria generate partially reduced sulfur and nitrogen compounds that can greatly affect the oxidation of metallic iron (31, 35). Shown in Fig. 3A to C are the formation and transformation of S and N intermediates by strain CVO in the presence of carbon steel coupons at an N/S ratio of 2.5 (results for other N/S ratios are shown in Fig. S4). CVO initially oxidized sulfide to biogenic S^0^ (Fig. 3A). Once sulfide was depleted, precipitated S^0^ was further oxidized to thiosulfate and sulfate (Fig. 3B). The oxidation of S species in this experiment was accompanied by reduction of 5.3 mM nitrate and the transient accumulation of 1.7 mM nitrite (Fig. 3C). Additional nitrite removal was via denitrification to N_2_, as has been previously shown for strain CVO (32).

Formation of biogenic S^0^ and thiosulfate was observed at all tested N/S ratios (Fig. S4). In addition, small amounts of sulfite (up to 84 μM) were detected at all N/S ratios (Table S1). Sterile sulfide-amended incubations did not show any measurable amounts of S^0^, sulfite, or thiosulfate (Fig. S1), demonstrating biogenic formations of those S intermediates by strain CVO. A lower N/S ratio shifted the accumulation of S species toward S^0^_,_ but thiosulfate was still detectable at the lowest tested N/S ratio of 0.4 (Fig. S4). This suggests that sulfide oxidation in strain CVO was incomplete, likely following equation 4 rather than equation 1.

(4) $$3{\text{HS}}^{-}+3.2\;{\text{NO}}_{3}^{-}+2.2\;{\text{H}}^{+}\to {\text{S}}_{2}{\text{O}}_{3}^{2-}+{\text{SO}}_{4}^{2-}+1.6\;{\text{N}}_{2}+2.6\;{\text{H}}_{2}\text{O,}\Delta {\text{G}}^{\text{0}\prime }=-491.6\;{\text{kJ mol}}^{-1}$$

### Time-resolved study of soNRB-induced corrosion.

Corrosion rates in Fig. 1A are aggregate mean values calculated from the metal weight loss that occurred over the entire 9-day experimental period. To better constrain corrosion kinetics in relation to metabolite profiles, a time-series experiment was conducted at an N/S ratio of 2.5, based on observations of high corrosion rates under these conditions (Fig. 1A). Corrosion rates increased in the early hours of the incubation and peaked at 0.45 mm y^−1^ after 24 h (Fig. 3D). This period of high corrosion coincided with the highest activity of strain CVO and accumulation of biogenic S^0^, nitrite, thiosulfate, and sulfate (Fig. 3A to C). Corrosion rates decreased to 0.14 mm y^−1^ as S^0^ and nitrite concentrations decreased. Parallel sterile incubations containing only sulfide and nitrate showed markedly lower corrosion rates, i.e., 0.2 mm y^−1^ during the first 24 h, dropping to 0.08 mm y^−1^ over the course of the 9 days (Fig. S5).

### Corrosiveness of the produced soNRB metabolites.

Strain CVO formed potentially corrosive metabolites, including S^0^, thiosulfate, and nitrite (24, 36–39). Yet, their simultaneous accumulation makes estimations of relative contributions to soNRB-induced MIC difficult. Therefore, the corrosion caused by individual N and S metabolites was assessed in sterile anoxic medium using concentrations comparable to those measured in CVO cultures. Because biogenic S^0^ can exhibit chemical alterations not seen in chemically produced S^0^ (e.g., thiol or organic groups) (40), biogenic S^0^ was harvested from CVO cultures for these tests.

**FIG 4:**
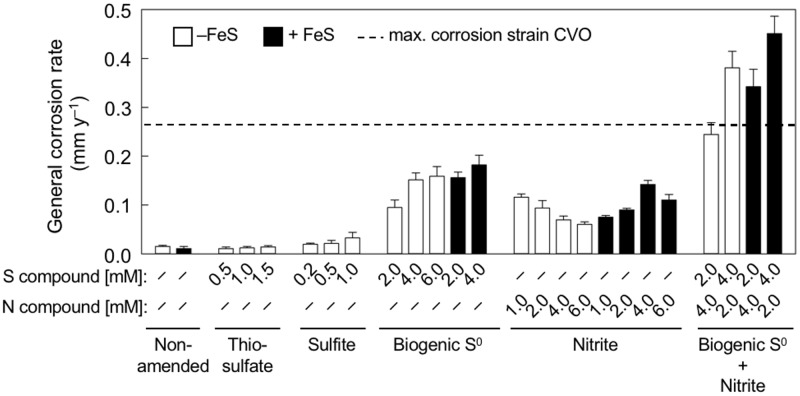
Corrosion rates determined by weight loss of carbon steel coupons after 9 days of exposure to sterile anaerobic medium with different concentrations of N and/or S species. Metal coupons were either directly exposed to individual species (white bars) or precorroded in sulfide-containing medium to create an iron sulfide (FeS) layer prior to the 9-day experimental incubations (black bars). Biogenic S^0^ refers to zero-valent sulfur harvested from cultures of strain CVO. For reference, the dashed line indicates the highest corrosion rate observed in incubations with strain CVO.

Increasing concentrations of biogenic S^0^ (2, 4, and 6 mM) caused corrosion at rates of 0.09 to 0.16 mm y^−1^, corresponding to about 35% to 59% of the maximum rates observed in CVO cultures (Fig. 4). Similar corrosion rates (0.07 to 0.12 mm y^−1^) were observed with nitrite (tested at 1, 2, 4, and 6 mM). Interestingly, corrosion was highest at the lowest tested concentration of nitrite (1 mM) and decreased with higher concentrations. Nitrate did not affect corrosion under any tested concentration (see sterile controls in Fig. 1A). Tests with 0.5 to 1.5 mM thiosulfate gave rise to corrosion rates that were similar to those in the absence of thiosulfate, and incubations with up to 1.0 mM sulfite showed only slightly increased corrosion rates of 0.03 mm y^−1^.

### Effect of iron sulfides on corrosion in the presence of S^0^ and nitrite.

Carbon steel pipelines carrying sulfidic produced waters or harboring sulfate-reducing bacteria will likely develop iron sulfides, such as mackinawite (FeS), as a corrosion product on internal steel surfaces (41–43). Previous reports attributed a catalytic role for FeS in corrosion mechanisms involving chemically prepared S^0^ (24, 25). Here, we investigated the effects of FeS in abiotic incubations with biologically generated (biogenic) S^0^, as well as with nitrite.

Corrosion rates measured on coupons with preformed FeS and biogenic 2 mM or 4 mM S^0^ were markedly higher than corrosion rates measured on bare steel surfaces (no FeS) with the same concentrations of biogenic S^0^ (Fig. 4). Nitrite, on the other hand, showed corrosion rates that were overall similar in range to FeS-free abiotic tests. On FeS-covered steel, however, an increase of nitrite concentrations also caused an increase in corrosion rates, which was contrary to the observations in FeS-free tests (Fig. 4).

Taken together, the results from abiotic experiments suggest that the formation of both biogenic S^0^ and nitrite increased corrosion in CVO cultures. Indeed, mixtures of S^0^ and nitrite achieved similarly high corrosion rates (0.24 to 0.44 mm y^−1^) as observed by strain CVO (Fig. 4), and their cumulative effect on corrosion might also explain the highest rate of up to 0.45 mm y^−1^ observed early in the time course experiment (Fig. 3D).

## DISCUSSION

### Towards a model for soNRB-mediated corrosion.

**FIG 5:**
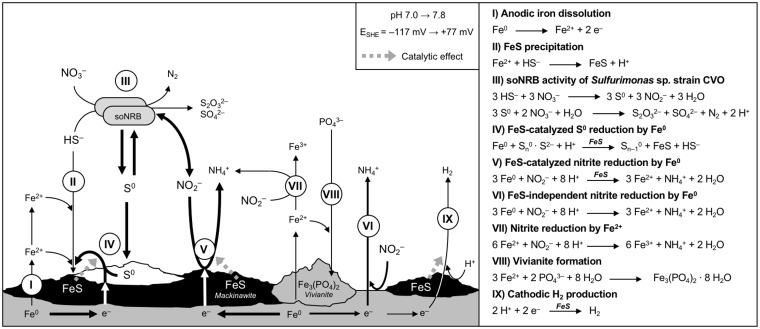
Schematic illustration of biotic and abiotic reactions at carbon steel surfaces in the presence of active soNRB, sulfide, and nitrate. Thickness of arrows indicates relative contribution to corrosion of the individual reactions. Dashed arrows indicate potential catalytic effects. Shift of pH and redox potential, as observed during growth of strain CVO in corrosion experiments, is also depicted. See Discussion for details.

In this study, we investigated corrosion caused by the soNRB Sulfurimonas strain CVO as a model for MIC in nitrate-treated oil field systems, particularly those experiencing produced water reinjection with elevated concentrations of dissolved sulfide. Insights obtained from biotic and abiotic corrosion tests in the present study are summarized in Fig. 5, offering a conceptual model for soNRB-mediated MIC. Details of this model are discussed below.

Sterile synthetic brines containing dissolved sulfide (1.5 to 10.8 mM) at neutral pH experienced only low corrosion of up to 0.04 mm y^−1^ (Fig. 1A). The main corrosion mechanism under these sterile conditions is the reaction of sulfide (H_2_S) with metallic iron (Fe^0^), which follows net equation 5 (42, 44):

(5) $$\begin{array}{ll} {\text{Fe}}^{0}+{\text{H}}_{2}\text{S}\to \text{FeS}+{\text{H}}_{2}, & \Delta {\text{G}}^{0}=-72.5\;{\text{kJ mol}}^{-1} \end{array}$$

The addition of the soNRB Sulfurimonas strain CVO increased corrosion rates by up to 7-fold, with severe pitting (Fig. 1 and 2). Whereas some sulfate-reducing bacteria and methanogenic archaea have been shown to accelerate corrosion kinetics directly by consuming cathodic electrons (electrical MIC or EMIC [2, 45–47]), strain CVO affected corrosion only indirectly, i.e., through the formation and excretion of corrosive metabolites (chemical MIC [CMIC]). MIC of carbon steel under the experimental conditions was quantitatively linked to the microbiological production of the corrosive metabolites S^0^ and nitrite (Fig. 5, reaction III). Indeed, mixtures of biogenic S^0^ and nitrite in abiotic experiments could reproduce the high corrosion rates associated with biological activity by strain CVO (Fig. 4). Neither the reduction of protons at FeS-covered steel surfaces in pH-neutral test medium (Fig. 5, reaction IX) nor the formation of thiosulfate and sulfite appeared to influence corrosion to any significant extent (Fig. 4).

In sour systems, such as sulfidic oil field brines, FeS readily forms on carbon steel surfaces (Fig. 5, reaction II [41, 48]) and plays a dual role in corrosion. FeS can form thin and tightly adhered films on steel surfaces (Fig. 5, reaction I) that act as a diffusion barrier for ferrous ions (Fe^2+^) and protect the underlying metal against corrosion (41–43). A similar passivation phenomenon was also observed here in sterile time course experiments (Fig. S5). FeS can also potentially contribute to corrosion by increasing the cathodic surface area, thereby accelerating proton reduction (Fig. 5, reaction IX [6, 49, 50]). In our experiments, FeS had another key role in corrosion, i.e., facilitating the cathodic reduction of steel-adsorbed biogenic S^0^ (Fig. 5, reaction IV), as evidenced by higher corrosion rates measured with FeS-covered steel surfaces (Fig. 4) and as seen elsewhere in experiments with chemically prepared S^0^ (24, 25). The cathodic reduction observed here was likely facilitated by reactive –SH groups present at FeS surfaces (51, 52). Furthermore, a direct comparison of biogenic S^0^ with chemically prepared S^0^ revealed similar corrosion rates (Fig. S6), underpinning the corrosiveness of S^0^ produced by strain CVO.

Nitrite was identified as another highly corrosive intermediate of CVO metabolism (Fig. 1 and 4). Nitrite can be reduced to ammonium abiotically with Fe^0^-derived electrons but also by oxidation of ferrous ions (Fig. 5, reactions VI and VII [37, 38, 53]). Owing to its ability to rapidly corrode steel, high concentrations of nitrite find technical application as a corrosion inhibitor in cooling water systems where nitrite quickly forms a layer of protective iron oxide corrosion products on internal steel surfaces (54–56). In agreement with this, corrosion rates decreased at higher nitrite concentrations in this study (Fig. 4). This trend appeared to reverse in the presence of a preformed FeS layer on the steel surface (Fig. 4). Iron sulfides have been shown to catalyze the reduction of nitrite to ammonium (Fig. 5, reaction V [57]), thereby likely influencing surface redox chemistry and ultimately corrosion rates when present on steel surfaces. Indeed, near-stoichiometric conversion of nitrite to ammonium was observed in abiotic experiments with and without FeS (Fig. S7). In addition to FeS, the iron phosphate mineral vivianite [Fe_3_(PO_4_)_2_·8H_2_O] was identified as an additional corrosion product in CVO cultures. Vivianite formation likely resulted from the high phosphate concentrations (1.5 mM) in the growth medium (58). In aqueous environments with low phosphate concentrations (e.g., oil field systems), the formation of vivianite as a corrosion product in the presence of nitrite would not be expected; in these settings, the aforementioned formation of iron oxides is more relevant (38, 59).

Starting concentrations of nitrate and sulfide (i.e., the N/S ratio) used in these experiments offer a useful proxy for testing different nitrate dosing regimens in oil reservoir-souring control scenarios. N/S ratios markedly affected corrosion in the presence of the soNRB strain CVO. Corrosion was related to the resulting level of partially reduced S and N metabolites (Fig. 1 and S4). Lower corrosion rates at an N/S ratio of 1.0 may be explained by shifting the metabolism of strain CVO to the formation of the noncorrosive products sulfate, thiosulfate, and N_2_ (equation 4) while limiting the accumulation of corrosive nitrite and biogenic S^0^ (Fig. S4).

### Practical considerations for soNRB-mediated MIC.

Carbon steel coupons exposed to sulfidic cultures of strain CVO corroded at rates of up to 0.27 mm y^−1^ (Fig. 1A; average weight loss) and caused pitting at linearly extrapolated rates of 2.4 mm y^−1^. The National Association of Corrosion Engineers (NACE) classifies such corrosion rates as severe (NACE SP0775-2013 [60]). Without any intervention, this type of MIC would challenge the integrity of oil field carbon steel infrastructure, which typically has a design life based on a corrosion allowance of ≤0.1 mm y^−1^, potentially leading to catastrophic failures.

It is anticipated that the extent of corrosion in a nitrate-treated PWRI system will vary and likely depend on prevailing physicochemical properties (e.g., temperature and water chemistry) and operating conditions of individual oil fields, as well as the particular microbial communities occurring in reservoirs and injection fluids. If dissolved sulfide is present in nitrate-treated produced waters, it is possible for soNRB to grow and cause severe corrosion, owing to their ability to form S^0^ and nitrite. The experiments presented here show that the N/S ratio could be a key factor governing such MIC, given that the ratio of electron acceptor to electron donor influences the types and concentrations of corrosive metabolites formed by soNRB. However, the activities of organotrophic NRB (11, 14, 15) and other nitrate-driven processes, such as FeS or Fe^2+^ oxidation (61, 62), likely proceed in parallel. Furthermore, it is possible that organotrophic NRB may even outcompete soNRB under certain conditions (63), so the prediction of proper nitrate dosing strategies in the field remains challenging.

Identifying S^0^ and nitrite as potential key drivers of corrosion begs the question of how this type of MIC might best be mitigated. The traditional approach to combatting oil field MIC is the periodic application of non-oxidizing biocides in order to control the growth and activity of corrosive biofilms (64). It is currently unknown whether such biocide treatments would also be effective against chemical MIC by soNRB. Another mitigation strategy may be the injection of sulfide scavengers into PWRI systems. Sulfide scavengers are used widely in oil field operations (65) and would remove the soNRB electron donor, hence preventing biogenic S^0^ accumulation. However, organotrophic NRB in oil field environments can produce corrosive nitrite through nitrate reduction coupled to the oxidation of organic electron donors that are abundant in produced waters (66–68), such that the removal of sulfide alone may not entirely control corrosion by all of the mechanisms presented here.

Overall, our results reveal that the oil field epsilonproteobacterium Sulfurimonas sp. strain CVO is capable of producing various corrosive S and N metabolites. This helps to explain the at times severe corrosion phenomena observed during nitrate-mediated souring control studies, in both laboratory and field trials (18–23). We identified several factors that contributed to soNRB-induced corrosion and offer mechanistic insights into this important type of CMIC (Fig. 5). Although effects during nitrate application in the field would likely vary due to biological and physicochemical properties in different reservoir and production systems, the results presented here point to ways to identify potential corrosion risks and implement suitable mitigation strategies.

## MATERIALS AND METHODS

### Strains and growth medium.

Sulfurimonas sp. strain CVO (NRRL B-21472) was obtained from the Agricultural Research Service Culture Collection (NRRL, USA). A simplified version of Coleville synthetic brine medium (CSB-A) without lactate was used to cultivate strain CVO (14). The medium contained, per liter of distilled water, 7.0 g NaCl, 0.2 g KH_2_PO_4_, 0.25 NH_4_Cl, 0.15 g CaCl_2_⋅2H_2_O, 0.4 g MgCl⋅6H_2_O, and 0.5 g KCl. The medium was autoclaved and cooled under an N_2_-CO_2_ (90:10) atmosphere before supplementing with 30 ml NaHCO_3_ (1 M), vitamins, trace elements, selenite, and tungstate solution and resazurin prepared as described before (69). The pH of the medium was adjusted to pH 7.0 to 7.1. The sterile anoxic medium was dispensed into autoclaved bottles, the headspace replaced with N_2_-CO_2_ (90:10), and the bottles sealed with butyl rubber stoppers. Sodium nitrate (NaNO_3_) and sodium sulfide (Na_2_S·9H_2_O) were added with sterile N_2_-flushed syringes from 2 M and 1 M sterile anoxic stock solutions, respectively.

For individual experiments, 5% (vol/vol) of freshly grown CVO cultures (with 3 mM sulfide and 10 mM nitrate) served as the inoculum. Strain purity was verified by microscopy and sequencing of the 16S rRNA gene.

### Preparation of biogenic zero-valent sulfur.

Biogenic S^0^ was harvested from 4 liters of actively growing cultures of strain CVO provided with 15 mM sulfide and 6 mM nitrate. Following sulfide depletion, the cultures were pasteurized for 30 min at 85°C and stored under an N_2_-CO_2_ (90:10) atmosphere at 4°C and overnight to promote sedimentation of S^0^. The supernatant was carefully discarded to remove cells and bulk medium. The precipitated S^0^ was washed three times in anoxic water and suspended in 100 ml anoxic CSB-A medium. The absence of cells was verified by microscopy. The resulting suspension was sealed and pasteurized under an N_2_ atmosphere for another 30 min at 85°C. For corrosion tests with commercially available sulfur (Merck Sigma-Aldrich, UK), a sulfur stock solution (200 mM) was prepared by suspending sulfur flowers in anoxic CSB-A medium and was pasteurized as described above. The concentration of S^0^ was determined by chloroform extraction and high-performance liquid chromatography (HPLC) (see below).

### Corrosion assessment.

General corrosion rates in CVO cultures were determined by weight loss analysis of carbon steel coupons (10 mm by 10 mm by 1 mm; EN 1.0330; Goodfellow Cambridge, UK). Coupons were treated in consecutive steps of submersion in 2 M HCl (<1 min), 1 M NaHCO_3_ (<10 s), deionized water (<10 s) and acetone (<10 s), dried under an N_2_ gas stream, and weighed on a microbalance. Before addition to the medium, the coupons were briefly sterilized in methanol, dried under N_2_, and placed in sterile plastic holders (Fig. S8A). After 9 days of incubation at room temperature, coupon weight loss was measured after the removal of corrosion products by the same cleaning procedure described above. Corrosion rates were expressed as metal thickness loss rate (in millimeters per year) and calculated from the weight loss value by considering the exposed surface area, density of carbon steel, and exposure time as described previously (46) from triplicate cultures grown under different nitrate-to-sulfide (N/S) ratios. Each of the three cultures contained three carbon steel coupons in a total volume of 200 ml. Sterile controls for all test conditions were incubated in the same way.

Time-dependent corrosion rate profiles were obtained from nine parallel sets of duplicate CVO cultures (100 ml), each containing 4 mM sulfide, 10 mM nitrate (N/S ratio, 2.5), and three carbon steel coupons. Duplicate cultures were sacrificed at nine consecutive time points over a total of 216 h (9 days) for weight loss determination from exposed coupons. Duplicate sterile incubations served as controls and were sacrificed at five out of the nine experimental time points.

Corrosion by specific metabolic intermediates was assessed in sterile, nitrate-free, and sulfide-free CSB-A medium (200 ml) in the same way as described above but without inoculation with strain CVO. Sulfite, thiosulfate, nitrite, or biogenic S^0^ was added from sterile anoxic stock solutions.

The effect of iron sulfides (FeS) on corrosion caused by the individual metabolic intermediates was assessed by exposing carbon steel coupons to 10 mM sulfide for 4 days in anoxic CSB-A medium. Inside a Coy anaerobic glove box (N_2_-H_2_ [95:5]), the coupons were then briefly rinsed in anoxic deionized water and transferred to fresh anaerobic CSB-A medium (200 ml). After the transfer, the bottle headspace was flushed with N_2_-CO_2_ (90:10), and the bottles were sealed with butyl rubber stoppers prior to the addition of individual chemical species. The average weight loss determined from coupons exposed to sulfide for 4 days was subtracted from the weight loss values determined at the end of the experiment before conversion to compound-specific corrosion rates.

### Pitting corrosion assessment.

In order to quantify localized corrosion by strain CVO, a separate experiment using specifically designed steel coupons and coupon holders was set up. Circular X52 carbon steel coupons were cleaned and preweighed as described above and inserted into customized coupon holders (1.25-cm^2^ exposure area) before addition to anoxic CSB-A medium in butyl rubber-stoppered 500-ml Duran bottles (see Fig. S8B). After adjusting the N/S ratios, the bottles were inoculated with 5% (vol/vol) of a fresh culture of strain CVO or directly incubated (sterile controls). The bottles were incubated horizontally at room temperature with the exposed coupon surface facing up.

After incubation, coupons were cleaned and stored in volatile corrosion inhibitor (VCI) paper (Protek Wrap, Daubert Cromwell, USA) in an N_2_ atmosphere until further analysis. For localized corrosion analysis, coupons were first scanned using a Keyence VR 3100 three-dimensional (3D) measurement macroscope (Keyence, USA) to visualize overall coupon topography. For higher-resolution imagery and quantification of pit depth, coupons were profiled by white-light interferometry using a ContourGT-X optical profiler (Bruker, Germany). The deepest localized pit (defined as a depressed region with average diameter of >20 μm) on each coupon was identified and measured.

### Chemical analysis.

Samples were taken with sterile N_2_-flushed syringes. The samples were directly analyzed or treated according to individual analytical requirements (see below) prior to storage at –20°C.

Dissolved sulfide concentrations were directly measured spectrophotometrically by the CuSO_4_ method, as described previously (70). Sulfide standards were prepared in anoxic deionized water using an anoxic stock solution of Na_2_S prepared as described by Widdel and Bak (69).

Nitrate, nitrite, and sulfate were quantified by ion chromatography using a Dionex ICS-1000 system equipped with an AS40 autosampler and an IonPac AS14A analytical column at 30°C. The eluent contained 8 mM Na_2_CO_3_ and 1 mM NaHCO_3_ at a flow rate of 1 ml/min.

Total zero-valent sulfur (S^0^) was extracted with chloroform from samples fixed with 5% (wt/vol) ZnCl_2_ solution and quantified as S_8_ on a Gilson HPLC system with a Gilson 118 UV-Vis detector (set to 263 nm) and a LiChrospher 100 reverse-phase (RP) C_18_ (125 by 4 mm, 5 μm) column (Merck Millipore, UK) at 20°C, as described before (71, 72). The eluent consisted of pure methanol at a flow rate of 1 ml/min. Sulfur standards were prepared by dissolving elemental sulfur (Sigma-Aldrich, UK) in chloroform.

For thiosulfate and sulfite quantification, samples were immediately derivatized with monobromobimane (MBB; Sigma-Aldrich, UK), as described previously (71). MBB-fixed samples were measured by ultrahigh-pressure liquid chromatography (UPLC) using a Waters Acquity H-class instrument with a Waters column (Acquity UPLC BEH C_8_, 1.7-μm, 2.1 by 50-mm column; Japan) and an acetic acid (0.25 [vol/vol] [pH 3.5])-methanol gradient flowing at 0.65 ml/min equipped with a Waters FLR fluorescence detector (excitation at 380 nm and absorbance at 480 nm).

Ammonium concentrations were determined spectrophotometrically by the Berthelot reaction, as described by López Pasquali and colleagues (73). A freshly prepared ammonium chloride solution served as a standard. Absorption was measured at 635 nm using ammonium-free deionized water as a blank.

The pH was measured with an Inlab Micro pH electrode (Mettler Toledo, UK), and the reduction-oxidation (redox) potential was measured with an Inlab Micro ORP electrode against an Ag/AgCl reference system (Mettler Toledo). The measured redox values (E_Ag/AgCl_) were converted to standard hydrogen electrode potentials (E_SHE_) and followed the equation E_SHE_ = E_Ag/AgCl_ + 222 mV.

### Scanning electron microscopy and corrosion product analysis.

Corroded steel coupons were retrieved from bottles in a Coy anaerobic glove box (N_2_-H_2_ [95:5]), briefly rinsed in anoxic water, and dried overnight inside the glove box. The coupons were kept under an N_2_ atmosphere until environmental scanning electron microscopy (ESEM). Coupon surfaces were analyzed with a FEI/Philips XL-30 field emission ESEM at 20 kV equipped with a Rontec Quantax thin window energy-dispersive X-ray (EDX) system. Due to overlapping signals of oxygen and nitrogen, the combined signal is reported here.

Mineral deposits on carbon steel coupons were analyzed with a PANalytical X’Pert Pro multipurpose diffractometer with Cu-K_α_ radiation operated at 40 kV and 40 mA. The data were collected in continuous mode over a range of 5 to 120° 2θ with a step size of 0.07° and nominal time per step of 500 s using the scanning X’Celerator detector. Phase identification was carried out with the PANalytical HighScore Plus with reference databases for identification of mineral phases.

## Supplementary Material

Supplemental file 1
