# Supplementary material for: Metabolites of an Oil Field Sulfide-Oxidizing, Nitrate-Reducing Sulfurimonas sp. Cause Severe Corrosion
Source: Appl Environ Microbiol. 2019 Jan 23;85(3):e01891-18. doi: 10.1128/AEM.01891-18 (PMC6344618; doi:10.1128/AEM.01891-18)
Supplement: Supplemental file 1 [file ad3fe89a38633ee17164a07acb01cbee_AEM.01891-18-s0001.pdf]

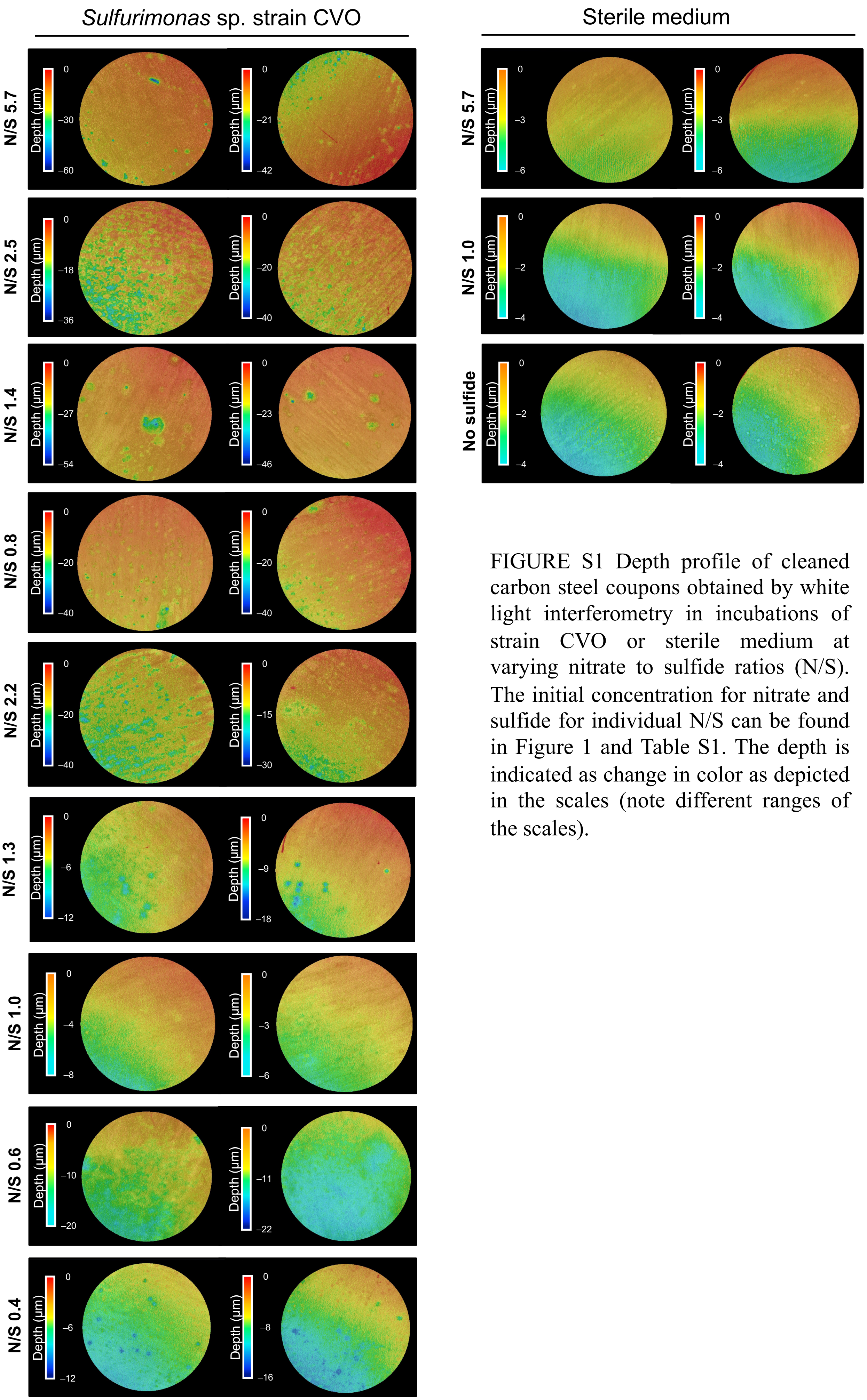

FIGURE S1 Depth profile of cleaned carbon steel coupons obtained by white light interferometry in incubations of strain CVO or sterile medium at varying nitrate to sulfide ratios (N/S). The initial concentration for nitrate and sulfide for individual N/S can be found in Figure 1 and Table S1. The depth is indicated as change in color as depicted in the scales (note different ranges of the scales).

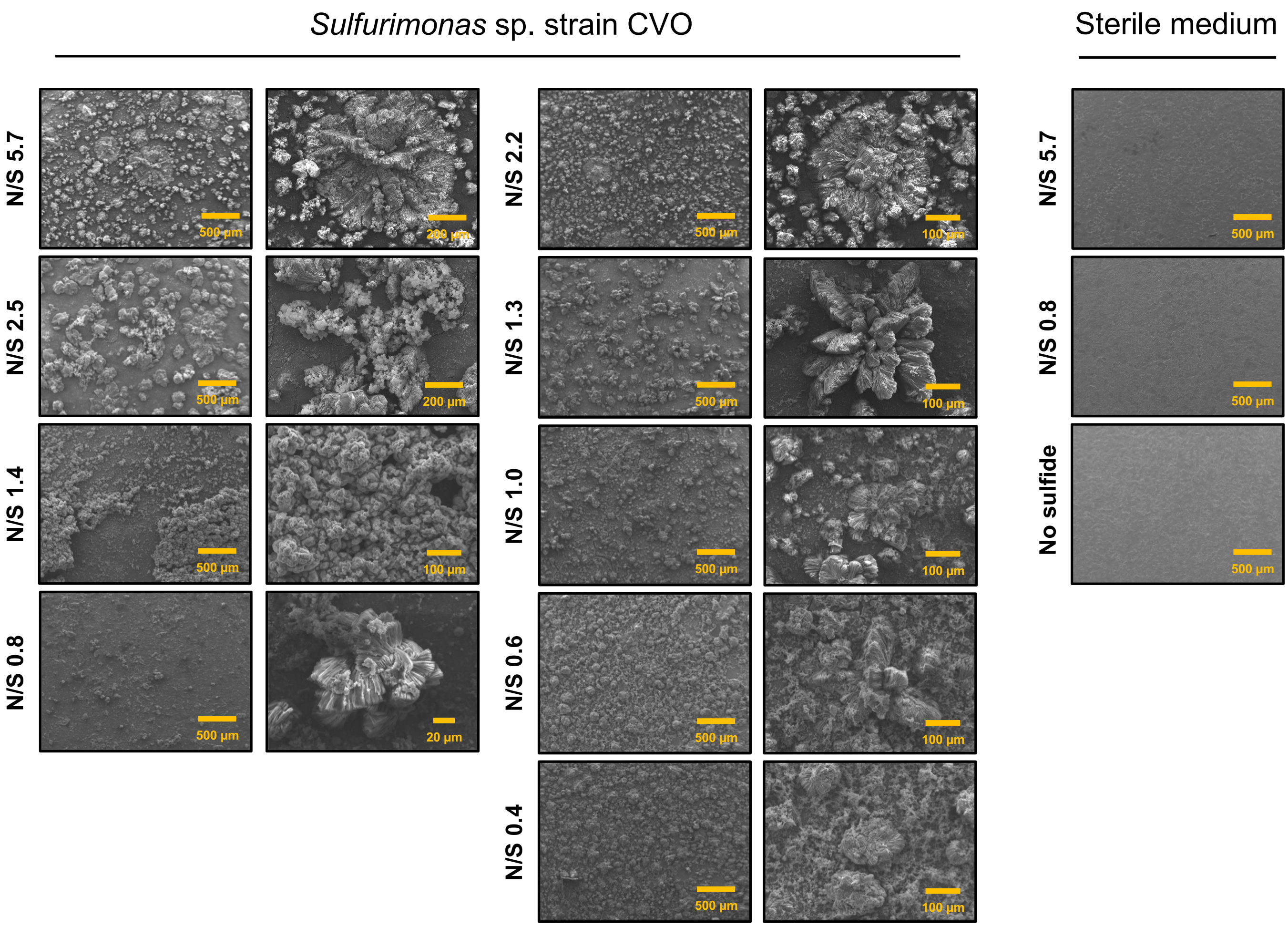

FIGURE S2 Scanning electron micro-graphs of carbon steel coupons in incubations of *Sulfurimonas* sp. strain CVO or sterile medium at varying nitrate to sulfide ratios (N/S). The initial concentration for nitrate and sulfide for individual N/S can be found in Figure 1 and Table S1. Images were recorded before and after removal of corrosion products. Information on the elemental composition of corrosion products (mineral and surfaces, as determined by energy-dispersive X-ray micro-analysis (EDX) can be found in Table S2.

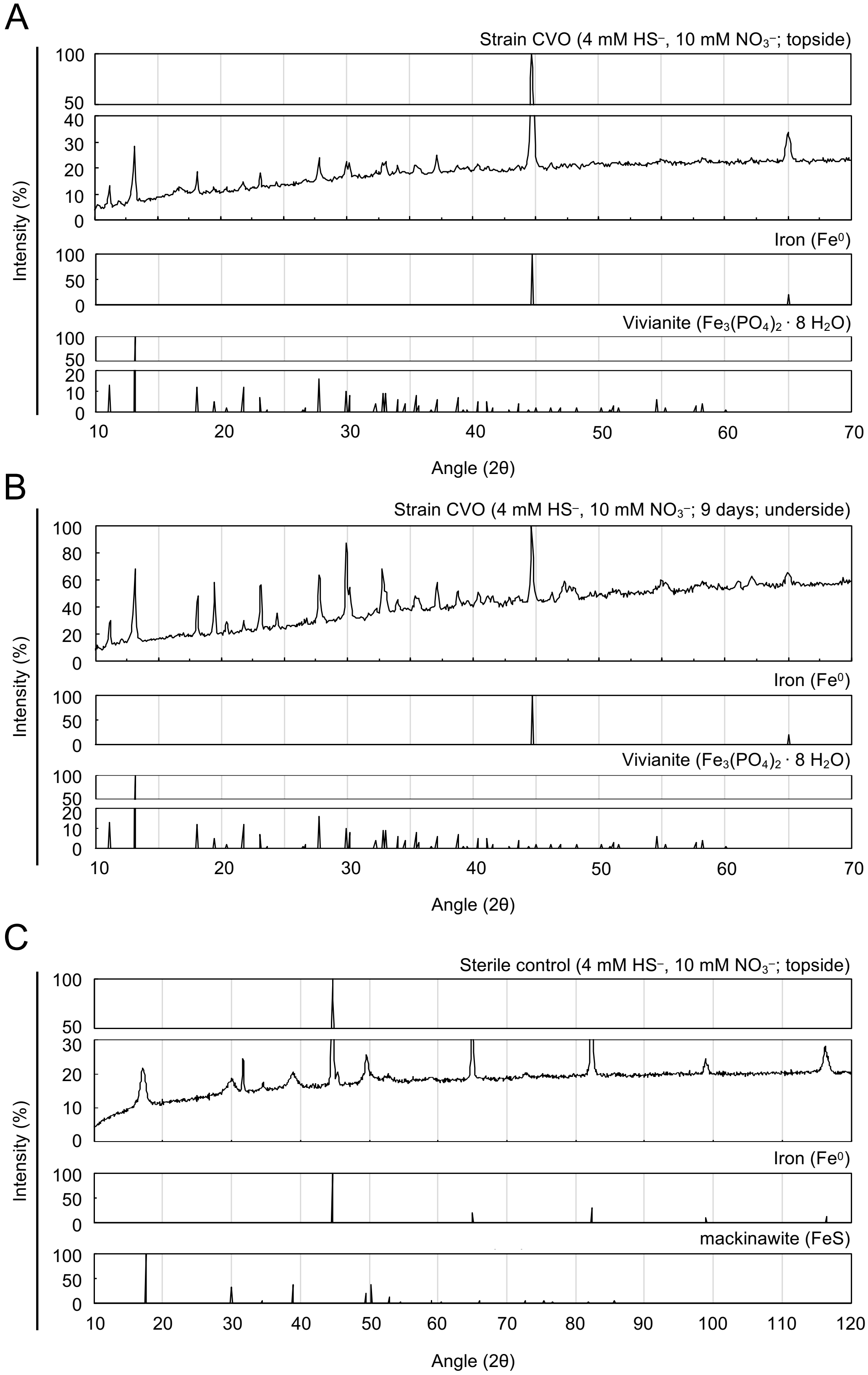

FIGURE S3 X-ray diffraction patterns for corrosion crust formed after 9 days on carbon steel coupons in incubation of strain CVO (A and B) or sterile medium (C). Reference patterns for identified compounds (iron, vivianite and mackinawite) are shown at the bottom. Axis breaks were introduced in certain plots for better visualization.

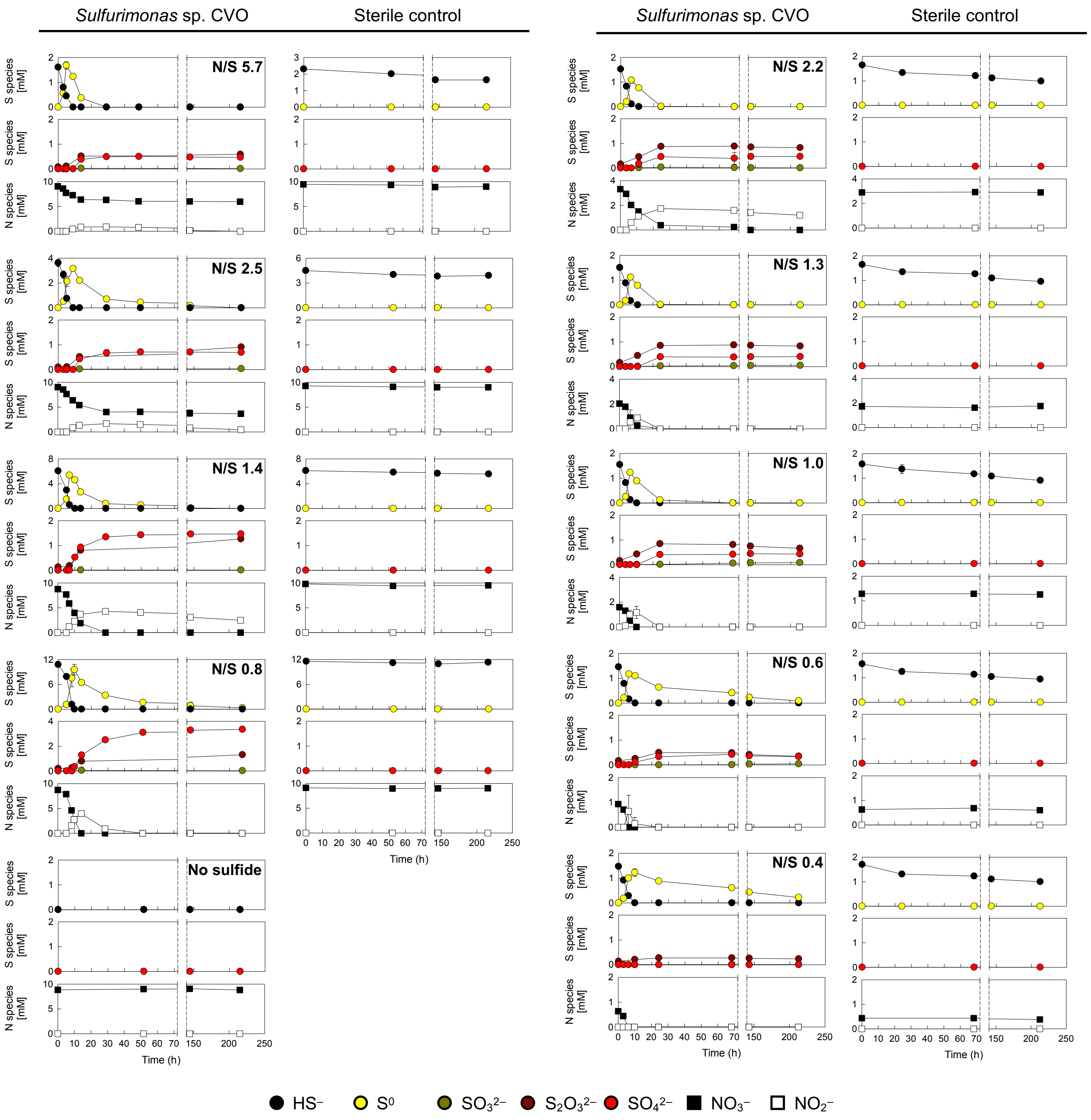

FIGURE S4 Changes in concentration of sulfur and nitrogen compounds in incubations of *Sulfurimonas* sp. strain CVO or sterile medium (see Figure 1 for corrosion rates). The applied nitrate to sulfide ratios (N/S) are indicated in the strain CVO plots.

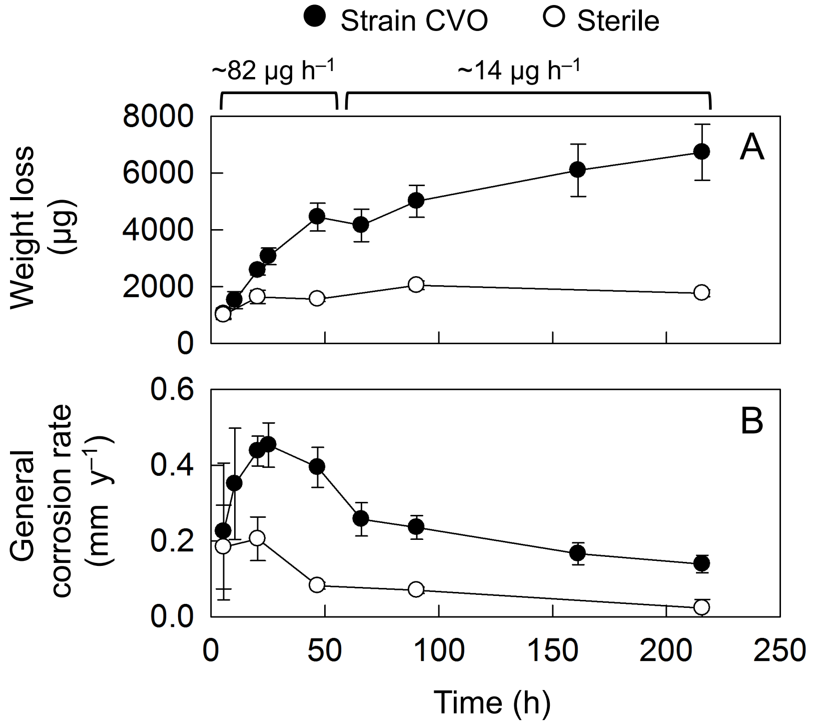

FIGURE S5 A) Weight loss profiles determined from carbon steel coupons exposed for different incubation times to cultures of *Sulfurimonas* sp. strain CVO or sterile medium. The initial concentrations of sulfide and nitrate were 4 mM and 10 mM, respectively. The rate of weight loss for CVO cultures were estimated by the linear increase in weight loss in two phases as indicated by the brackets above the graph. B) Corresponding corrosion rate profiles determined from weight loss values (see materials and methods for details). The CVO corrosion profile in B) was used to plot corrosion rates alongside concentration of individual N and S compounds in Figure 3.

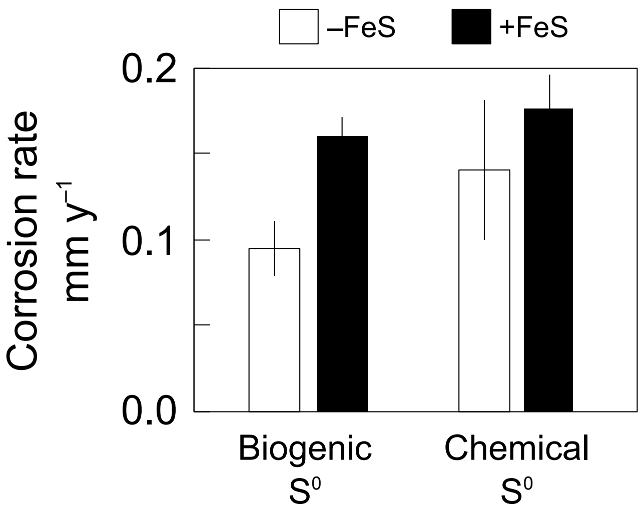

FIGURE S6 Corrosion rates determined by weight loss of carbon steel coupons after nine days of exposure to sterile anaerobic medium with 2 mM biogenic zero-valent sulfur (S^0) from strain CVO or 2 mM chemically produced commercial S^0. Metal coupons were either directly exposed (white bars) or pre-corroded in sulfide containing medium to create an iron sulfide (FeS) layer prior to the nine days experimental incubations (black bars).

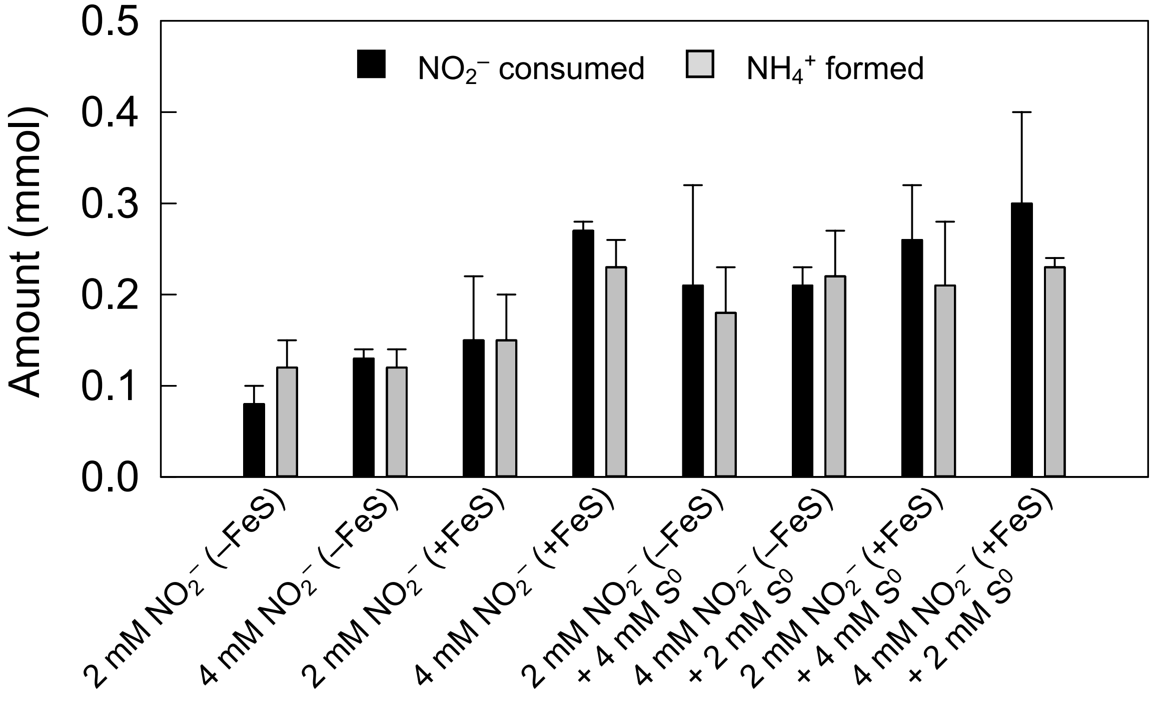

FIGURE S7 Comparison between amounts of nitrite (NO_2^-) consumed and amounts of ammonium formed in abiotic incubations with (+FeS) or without (–FeS) pre-formed iron sulfide layers on carbon steel coupons (see Fig. 4 for respective corrosion rates). Changes in ammonium were <0.04 mmol when coupons or nitrite were omitted (not shown).

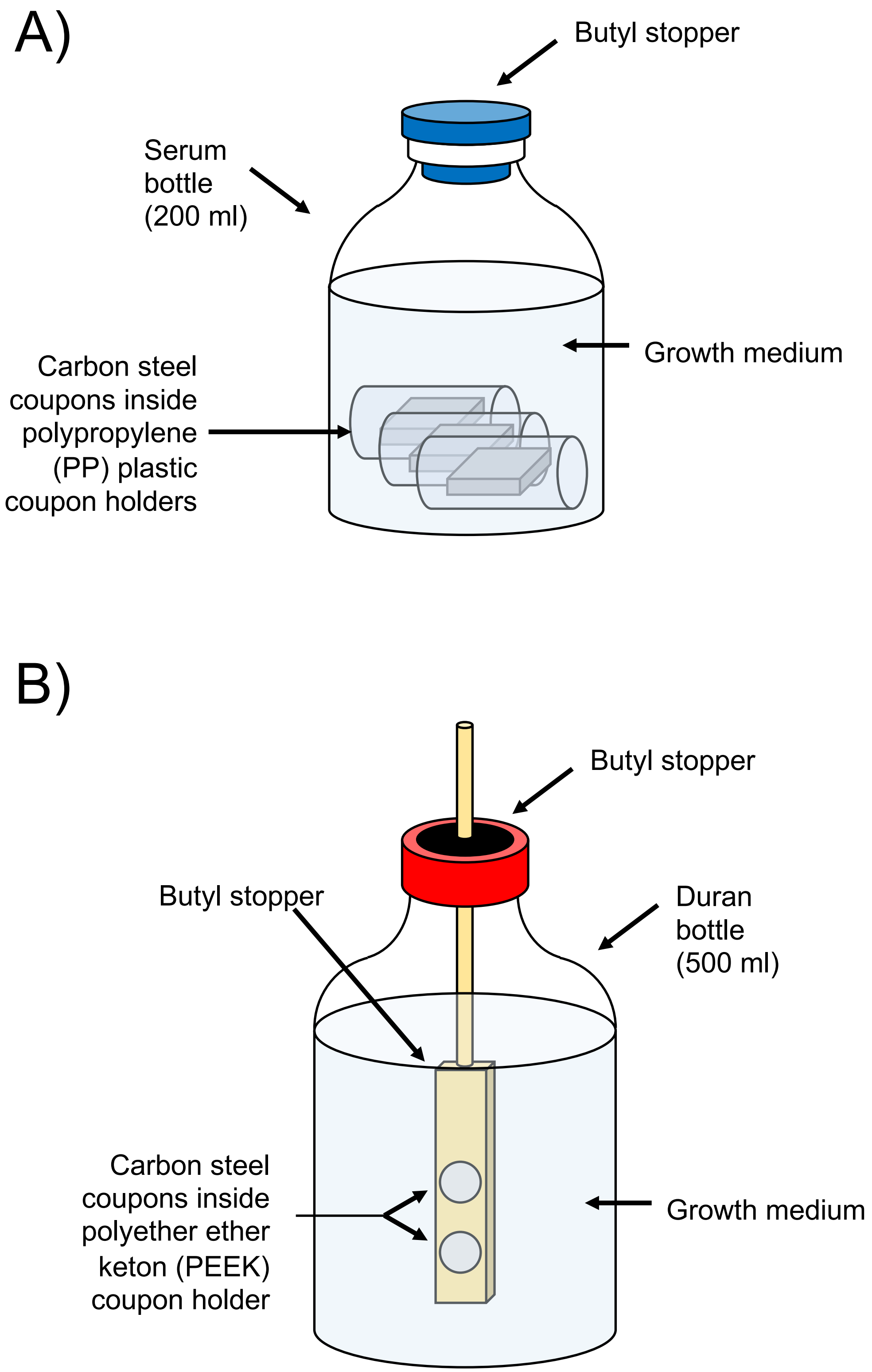

FIGURE S8 Experimental set-up for the assessment of general corrosion rates (A) pitting corrosion (B). Further details can be found in the material and method section. Coupon holders in A were prepared by cutting out a 2 cm long mid-section from a 2 ml micro-centrifuge tube.

Table S1 Summary of key parameters of individual incubations of *Sulfurimonas* sp. strain CVO.

N/S ratio	Initial nitrate	Final amount of nitrate	Maximal amount nitrite formed	Final amount nitrite	Initial sulfide	Maximal amount zero-valent sulfur formed	Final amount zero-valent sulfur	Maximal intermediate amount sulfite	Final amount thiosulfate	Final amount sulfate	Initial pH	Final pH	Initial redox potential (E_{SHE})	Final redox potential (E_{SHE})	General corrosion rate	Maximal pit depths detected
	[mM]	[mM]	[mM]	[mM]	[mM]	[mM]	[mM]	[µM]	[mM]	[mM]			(mV)	(mV)	(mm y^{-1})	(µm)
Strain CVO (varying sulfide concentration):																
**5.7**	9.1 (0.2)	6.0 (0.2)	**0.9 (0.2)**	**0.0 (0.0)**	1.6 (0.1)	**1.7 (0.2)**	**0.0 (0.0)**	26 (0)	**0.6 (0.0)**	0.5 (0.0)	7.03 (0.03)	7.63 (0.08)	–59 (11)	44 (10)	**0.23 (0.02)**	60 / 42
**2.5**	9.0 (0.3)	3.7 (0.4)	**1.7 (0.3)**	**0.4 (0.4)**	3.6 (0.3)	**3.2 (0.2)**	**0.0 (0.0)**	40 (2)	**0.9 (0.1)**	0.7 (0.0)	7.01 (0.05)	7.57 (0.05)	–83 (8)	47 (13)	**0.26 (0.01)**	31 / 31
**1.4**	8.8 (0.2)	0.0 (0.0)	**4.3 (0.2)**	**2.5 (0.2)**	6.1 (0.2)	**5.4 (0.4)**	**0.0 (0.0)**	13 (2)	**1.3 (0.1)**	1.5 (0.0)	7.14 (0.04)	7.80 (0.11)	–107 (3)	43 (5)	**0.27 (0.03)**	54 / 46
**0.8**	8.7 (0.2)	0.0 (0.0)	**4.0 (0.4)**	**0.0 (0.0)**	10.8 (0.2)	**9.6 (1.3)**	**0.3 (0.5)**	52 (0)	**1.3 (0.1)**	3.4 (0.1)	7.08 (0.06)	7.78 (0.03)	–117 (2)	12 (9)	**0.14 (0.02)**	39 / 34
Strain CVO (varying nitrate concentration):																
**2.2**	3.3 (0.1)	0.0 (0.0)	**1.7 (0.1)**	**1.2 (0.1)**	1.5 (0.0)	**1.1 (0.1)**	**0.0 (0.0)**	36 (0)	**0.8 (0.0)**	0.5 (0.0)	7.01 (0.01)	7.46 (0.09)	–71 (3)	59 (4)	**0.20 (0.02)**	35 / 19
**1.3**	2.0 (0.1)	0.0 (0.0)	**0.9 (0.1)**	**0.0 (0.0)**	1.5 (0.1)	**1.1 (0.0)**	**0.0 (0.0)**	49 (1)	**0.8 (0.0)**	0.4 (0.0)	7.01 (0.02)	7.36 (0.02)	–81 (2)	73 (2)	**0.12 (0.02)**	17 / 11
**1.0**	1.6 (0.0)	0.0 (0.0)	**1.5 (0.5)**	**0.0 (0.0)**	1.6 (0.1)	**1.2 (0.1)**	**0.0 (0.0)**	84 (1)	**0.7 (0.1)**	0.4 (0.0)	7.00 (0.01)	7.27 (0.02)	–84 (5)	77 (2)	**0.09 (0.01)**	7 / 5
**0.6**	0.9 (0.1)	0.0 (0.0)	**0.6 (0.6)**	**0.0 (0.0)**	1.5 (0.0)	**1.2 (0.0)**	**0.1 (0.1)**	43 (3)	**0.4 (0.1)**	0.3 (0.0)	7.01 (0.02)	7.30 (0.02)	–88 (6)	72 (6)	**0.13 (0.01)**	22 / 19
**0.4**	0.6 (0.1)	0.0 (0.0)	**0.0 (0.0)**	**0.0 (0.0)**	1.5 (0.0)	**1.2 (0.2)**	**0.2 (0.0)**	8 (0)	**0.2 (0.0)**	0.0 (0.0)	7.00 (0.01)	7.32 (0.01)	–90 (3)	70 (7)	**0.14 (0.01)**	15 / 11

Values in brackets refers to standard deviation

Table S2 Elemental composition determined by EDX of corrosion products formed on carbon steel coupons during incubations with *Sulfurimonas* sp. strain CVO.

		Sulfurimonas sp. strain CVO									Sterile medium		
**Structure**	**Elements**	**Normalized amount (wt %)**	**Normalized amount (wt %)**	**Normalized amount (wt %)**	**Normalized amount (wt %)**	**Normalized amount (wt %)**	**Normalized amount (wt %)**	**Normalized amount (wt %)**	**Normalized amount (wt %)**	**Normalized amount (wt %)**	**Normalized amount (wt %)**	**Normalized amount (wt %)**	**Normalized amount (wt %)**
	**N/S ratio**	**5.7**	**2.5**	**1.4**	**0.8**	**2.2**	**1.3**	**1.0**	**0.6**	**0.4**	**N/A**	**5.7**	**0.8**
**Mineral**	Fe	38.72	36.30	37.52	32.81	35.76	36.46	36.68	35.70	39.33	N/A	N/A	N/A
	P	12.03	10.78	8.15	8.46	9.43	8.09	8.26	8.32	8.03	N/A	N/A	N/A
	S	0.00	0.00	0.00	4.92	0.00	0.70	1.20	1.64	2.41	N/A	N/A	N/A
	O/N	47.20	52.92	47.15	51.02	54.81	52.64	51.85	52.88	48.49	N/A	N/A	N/A
**Surface layer**	Fe	77.49	59.24	59.30	60.96	76.52	72.41	64.59	70.74	69.71	100.00	65.75	77.32
	P	1.88	2.71	3.22	4.14	1.46	0.00	3.15	0.00	2.74	0.00	0.00	0.00
	S	7.42	12.32	13.77	25.36	8.72	14.32	25.70	26.20	22.66	0.00	34.25	22.68
	O/N	12.23	25.74	22.78	8.62	13.31	13.27	6.56	3.05	3.96	0.00	0.00	0.00
